# Supplementary material for: Soil Mite (Acari: Mesostigmata) Communities and Their Relationships with Some Environmental Variables in Experimental Grasslands from Bucegi Mountains in Romania
Source: Insects. 2022 Mar 14;13(3):285. doi: 10.3390/insects13030285 (PMC8953817; doi:10.3390/insects13030285)
Supplement: Supplementary file 1 [file insects-13-00285-s001.zip › insects-1620589-supplementary.pdf]

Soil mite communities (Acari: Mesostigmata) and their relationship with some environmental variables in experimental grasslands from Bucegi Mountains in Romania

Insects

Manu M.[1†], Băncilă R.I.[2,3], Mountford O.[4], Maruşca T.[5], Blaj V.A.[5], Onete M.[1†]

[1]Romanian Academy, Institute of Biology Bucharest, Department of Ecology, Taxonomy and Nature Conservation, street Splaiul Independenţei, no. 296, zip code 0603100, PO-BOX 56-53, fax 040212219071, tel. 040212219202, Bucharest, Romania, email: minodoramanu@gmail.com and marilena.onete@gmail.com

[2]Faculty of Natural Sciences, University Ovidius Constanţa, Constanţa, Romania

[3]Department of Biospeleology and Soil Edaphobiology, "Emil Racoviţă" Institute of Speleology, Romanian Academy, 13 Septembrie Road, No. 13, 050711, Bucharest, Romania, bancila_ralucaioana@yahoo.com

[4]Centre for Ecology and Hydrology, Maclean Building, Benson Lane, Crowmarsh Gifford, Wallingford, Oxfordshire, OX10 8BB, UK, email: om@ceh.ac.uk

[5]Grassland Research and Development Institute Braşov, Romania, Cucului str., No. 5, 500128, Braşov,
Romania, email: maruscat@yahoo.com; blajadi@pajisti-grassland.ro

Corresponding author: minodoramanu@gmail.com and marilena.onete@gmail.com
† Authors with equal contribution in the production of the article

Table S1. Numerical abundance (Ab.) and frequency (F%) of Mesostigmata mites from experimental plots, 2017.

Species	Code	CG		A		B		C		D		Total	
		Ab.	F%	Ab.	F%	Ab.	F%	Ab.	F%	Ab.	F%	Ab.	F%
Alliphis haleri (G. & R.Canestrini, 1881)	Allihale									9	6	9	6
Arctoseius cetratus (Sellnick, 1940)	Arctcert	38	20	37	28	17	18	42	26	176	52	310	90
Arctoseius insularis (Willmann, 1952)	Arctinsu							13	4	6	2	19	6
Cheroseius bryophilus Karg, 1969	Cherbryo							2	4	62	16	64	20
Dendrolaelaps foveolatus (Leitner, 1949)	Dendfove							118	6	96	6	214	12
Dendrolaelaps sp.	Dendsp					17	8	4	6	4	4	25	18
Dinychus carinatus Berlese, 1903	Dinyca							2	4	6	4	8	8
Dinychus sp.	Dinysp									9	14	9	14
Gamasellodes insignis (Hirschmann, 1963)	Gamainsi									10	2	2	2
Gamasellodes bicolor (Berlese, 1918)	Gamabico									2	2	10	2
Geolaelaps nolli (Karg, 1962)	Geolnoll	3	6	85	30	8	6	31	18	65	18	192	64
Geolaelaps praesternalis Willmann, 1949	Geolprae	1	2	27	4					3	6	31	10
Iphidozercon gibbus (Berlese, 1903)	Iphigibb					3	6			4	2	7	6
Leioseius magnanalis (Evans, 1958)	Leiomagn									1	2	1	2
Lysigamasus conus Karg, 1971	Lysiconu	3	6	2	4	1	2	22	14	63	32	91	42
Neopodocinum mrciaki Selinick, 1968	Neopmrch									2	4	79	32
Mixozercon sellnicki (Schweizer 1948)	Mixosell	5	8	41	18	3	2	30	10			2	4
Olopachys vysotskajae Koreleva, 1976	Olovyso			6	2							6	2
Onchodellus sp.	Onchsp									4	4	4	4
Onchodellus karawaiewi Berlese, 1920	Onchkara			1	2							1	2
Oodinychus sp.	Oodisp									1	2	1	2
Pergamasus laetus Juvara-Bals, 1970	Perglaet			1	2			9	6	9	14	19	18
Pergamasus norvegicus (Berlese, 1906)	Pergnorv									10	10	10	12
Pergamasus quisquiliarum (Canestrini & Canestrini, 1882)	Pergquis									2	4	2	4

Prozercon sp.	Prozsp					13 2	13	2
Pseudoparasitus sellnicki (Bregetova & Koroleva, 1964)	Pseusell					3 6	3	6
Uroobovella sp.	Uroosp			3 6		19 10	22	16
Veigaia planicola Berlese, 1892	Veigplan					1 2	4	2
Veigaia nemorensis (C.L.Koch, 1836)	Veignemo					4 2	1	2
Vulgarogamasus kraepelini (Berlese, 1904)	Vulgkrae		4 2				4	2
Total number of species		5	9	7	10	26	30	
Total no. of individuals		50	204	52	273	584	1163	
